# Efficient Simultaneous Isolation of Pinostrobin and Panduratin A from *Boesenbergia rotunda* Using Centrifugal Partition Chromatography

**DOI:** 10.3390/molecules29215186

**Published:** 2024-11-02

**Authors:** Wanna Eiamart, Supeecha Wittayalertpanya, Sarin Tadtong, Weerasak Samee

**Affiliations:** 1 Department of Pharmaceutical Chemistry, Faculty of Pharmacy, Srinakharinwirot University, Nakhon Nayok 26120, Thailand; wanna.eiamart@g.swu.ac.th; 2 Chula Pharmacokinetic Research Center, Faculty of Medicine, Chulalongkorn University, Bangkok 10330, Thailand; supeechas@hotmail.com; 3 Center of Excellence in Clinical Pharmacokinetics and Pharmacogenomics, Faculty of Medicine, Chulalongkorn University, Bangkok 10330, Thailand; 4 Department of Pharmacognosy, Faculty of Pharmacy, Srinakharinwirot University, Nakhon Nayok 26120, Thailand; sarin@g.swu.ac.th

**Keywords:** extraction, pinostrobin, panduratin A, *Boesenbergia rotunda*, centrifugal partition chromatography, purification, mass spectrometry

## Abstract

The bioactive flavonoids pinostrobin (PN) and panduratin A (PA) from *Boesenbergia rotunda* are essential for research and therapeutic applications. This study introduces an innovative method utilizing ultrasound-assisted extraction with *n*-hexane pre-treatment, followed by one-step centrifugal partition chromatography (CPC) purification. Extraction efficiency was evaluated using ultra high-performance liquid chromatography (UHPLC), and the isolated compounds were characterized through ^1^H-NMR and liquid chromatography electrospray ionization tandem mass spectrometry (LC-ESI-MS/MS), adhering to AOAC validation guidelines. Optimal extraction conditions comprised a particle size of 125 μm, a solid-to-liquid ratio of 1:30 g/mL, and a 10 min extraction time, yielding a crude extract of 6.96 ± 0.07%. Using an *n*-hexane/MeOH/water (5/3.4/1.6, *v*/*v*) solvent system in ascending mode, PN (2.16 mg, 98.78% purity) and PA (0.4 mg, 99.69% purity) were isolated from 67 mg of crude extract within 30 min. This streamlined approach enhances purification efficiency, allowing for faster extraction and higher purity, making it a suitable method for commercial applications.

## 1. Introduction

*Boesenbergia rotunda*, known as “krachai” in Thailand, belongs to the Zingiberaceae family and is highly regarded for its extensive use in traditional medicine, with potential applications as both a medicinal and food supplement. This plant is characterized by a wide array of biological and pharmacological properties, which have garnered significant research interest. Traditionally, it has been employed in the treatment of various ailments, including rheumatism, muscle pain, fever, gastrointestinal disorders, flatulence, dyspepsia, and peptic ulcers, with its rhizome being a critical component in many traditional Thai medicinal formulations [1,2,3]. The pharmacological importance of *B. rotunda* is primarily due to its rich phytochemical composition, encompassing flavonoids and essential oils. Research has identified over 20 flavonoid compounds in this plant, mainly categorized into flavanones and chalcones, which are responsible for its therapeutic effects [4,5]. For instance, compounds such as pinostrobin and panduratin A exhibit anti-inflammatory and antioxidant properties, essential for combating oxidative stress and conditions related to inflammation [5,6]. Studies have also demonstrated the efficacy of *B. rotunda* extracts in alleviating symptoms associated with diabetes and neuropathic pain, underscoring its potential as a natural remedy for chronic ailments [6,7]. The safety and therapeutic effectiveness of *B. rotunda* are supported by numerous studies which have thoroughly evaluated its biological activities. For instance, a randomized controlled trial validated its effectiveness in treating functional dyspepsia, further supporting its traditional applications [2]. Additionally, *B. rotunda* extracts have shown protective effects against doxorubicin-induced cardiotoxicity, indicating its potential use as an adjunct therapy in cancer treatment [8]. The presence of bioactive compounds such as flavonoids and essential oils not only contributes to its medicinal properties, but also emphasizes the significance of *B. rotunda* in the domain of functional foods and nutraceuticals [3,9]. PA and PN are prominent bioactive compounds extracted from *B. rotunda*, attracting significant interest in medicinal research due to their diverse therapeutic potentials. These compounds have been extensively investigated for their antioxidant, anti-inflammatory, anti-tumor, antibacterial, anti-obesity, and anti-cancer properties [10,11,12]. For example, PA has been shown to activate AMP-activated protein kinase (AMPK), a crucial enzyme in regulating lipid metabolism, thereby offering potential benefits for obesity management [10]. Moreover, both PA and PN have demonstrated protective effects against oxidative stress and inflammation, which are critical contributors to various chronic diseases [11,12].

The isolation and purification of bioactive compounds from *B. rotunda* traditionally utilize methods such as silica gel chromatography, Sephadex-LH 20 column chromatography, and preparative high-performance liquid chromatography (HPLC) [5,13,14]. Despite their widespread use, these techniques are often time-consuming and require large amounts of starting material. Additionally, the target compounds may become irreversibly adsorbed to the stationary phase during separation, complicating the purification process [5,13]. Notably, current methodologies have not achieved the simultaneous isolation of both PA and PN, revealing a significant gap in existing techniques [5,12].

To address these challenges, preparative centrifugal partition chromatography (CPC) is proposed as a viable alternative that can effectively overcome the limitations of traditional purification techniques. This method allows for the simultaneous purification of multiple compounds while minimizing the risk of irreversible adsorption [5]. Notably, the application of CPC for the separation of PA and PN has not been previously explored, indicating a novel research avenue that could enhance the efficiency of isolating these valuable compounds from *B. rotunda* [15,16,17]. Given the therapeutic benefits associated with these flavonoids in treating various diseases, it is imperative to develop new isolation and purification methodologies utilizing CPC to address the shortcomings of conventional methods. Moreover, to further enhance the efficiency of isolate development, ultra high-performance liquid chromatography with ultraviolet detection (UHPLC-UV) serves as a powerful analytical technique for quantifying isolated compounds, especially in natural products [15,16]. For precise identification and quantification of compounds within complex mixtures, liquid chromatography electrospray ionization tandem mass spectrometry (LC-ESI-MS/MS) is employed to detect a variety of bioactive compounds, including PN and PA in *B. rotunda* [18,19,20,21]. Consequently, this study aims to establish an efficient method for purifying these active compounds from *B. rotunda* using CPC, with subsequent quantification and characterization assessed through UHPLC-UV and LC-ESI-MS/MS, thereby paving the way for their potential therapeutic applications.

## 2. Results and Discussion

### 2.1. Validation of Method for Quantification PN and PA

The analytical method employed in this study was rigorously validated for critical parameters including selectivity, linearity, precision, recovery, limit of detection (LOD), and limit of quantitation (LOQ), adhering to the guidelines set forth by the AOAC [17].

#### 2.1.1. Validation of UHPLC-UV Method for Quantification PN and PA in *B. rotunda* Crude Extract

**Figure 1:**
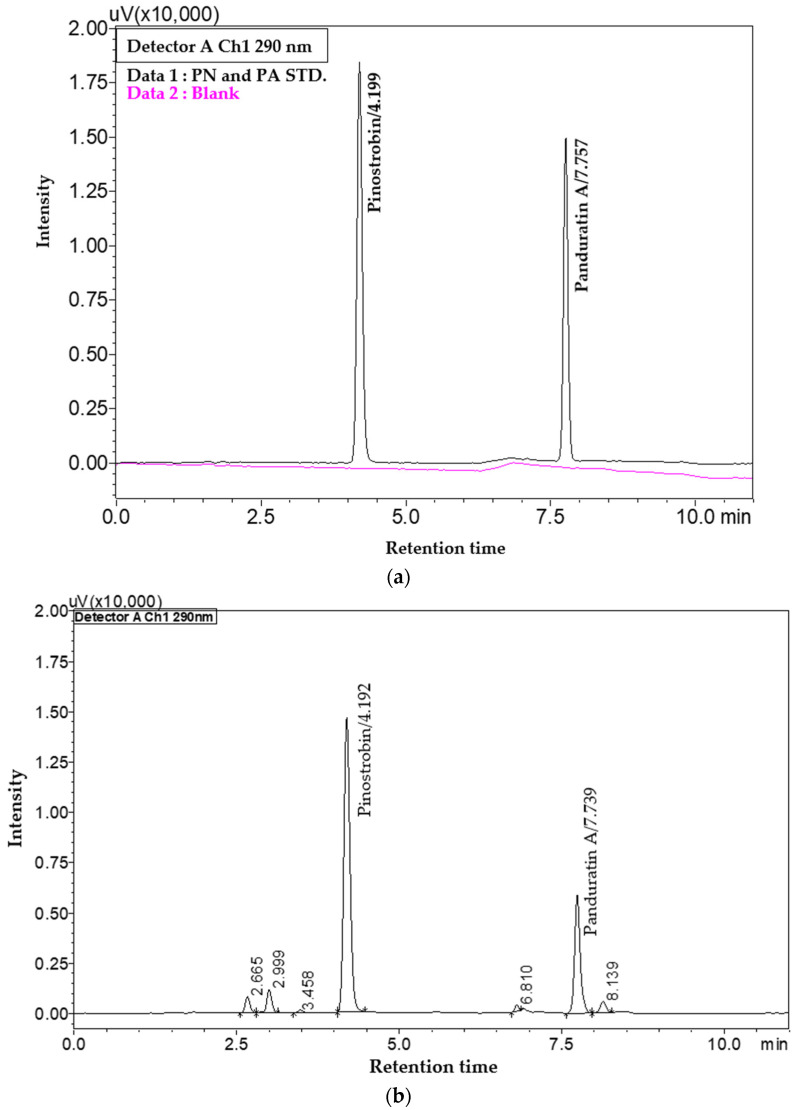
RP-HPLC chromatogram of (**a**) overlaid chromatogram of blank (DMSO in MeOH) compared with standard solution at 10 µg/mL and (**b**) crude *n*-hexane extract of *B. rotunda* at 12.5 µg/mL was spiked standard solution at 2.5 µg/mL.

PN and PA were detected at retention times of 4.19 and 7.75 min, respectively. Selectivity was confirmed as the method showed no endogenous interference at the elution times of PN and PA in the sample diluent, which is crucial for ensuring the accuracy of the results. Representative chromatograms of blank and standard solution are illustrated in Figure 1a, further supporting the method’s selectivity. The calibration curves for both PN and PA demonstrated exceptional linearity, with R^2^ values exceeding 0.999 across three replicates, as shown in Table 1, indicating a robust method suitable for quantitative analysis. The LOD and LOQ were approximated by calculating from the calibration plot, employing the following formulas [22,23]:

LOD = 3.3 × (Standard Deviation of Y-intercept)*/*(Mean of Slope)

LOQ = 10 × (Standard Deviation of Y-intercept)*/*(Mean of Slope)

which were 0.042 µg/mL and 0.126 µg/mL for PN and 0.044 µg/mL and 0.133 µg/mL for PA, respectively. Both intra- and inter-day precision were evaluated by analysis of six independent samples and three consecutive validation days, with the overall percentage relative standard deviation (%RSD) being less than 2 for all samples (Table 1), indicating high precision and reliability of the method. Recovery studies are shown in Figure 1b and Table 1 based on three determinations (crude *n*-hexane extract sample was spiked standard at 0.65, 2.5 and 10 µg/mL) and three replicates. The %recovery results range from 100.06 ± 0.96% to 102.19 ± 0.30% for PN and from 101.08 ± 0.49% to 102.95 ± 2.01% for PA, demonstrating the method’s accuracy and consistency and confirming its suitability for quantitative analysis of PN and PA in *B. rotunda* extract. The findings align with previous studies that have established similar validation parameters for analytical methods, reinforcing the credibility of the current method [18]. Overall, the validation results underscore the impressive nature of this method, demonstrating its potential for analyzing PN and PA in complex mixtures such as *B. rotunda* crude extract. Moreover, another remarkable feature is the drastically reduced analysis time of just 11 min, a significant improvement compared to the previous studies that required as much as 50 min [5,19].

#### 2.1.2. Validation of LC-ESI-MS/MS Method for Quantification PN and PA of Purified Compounds

**Figure 2:**
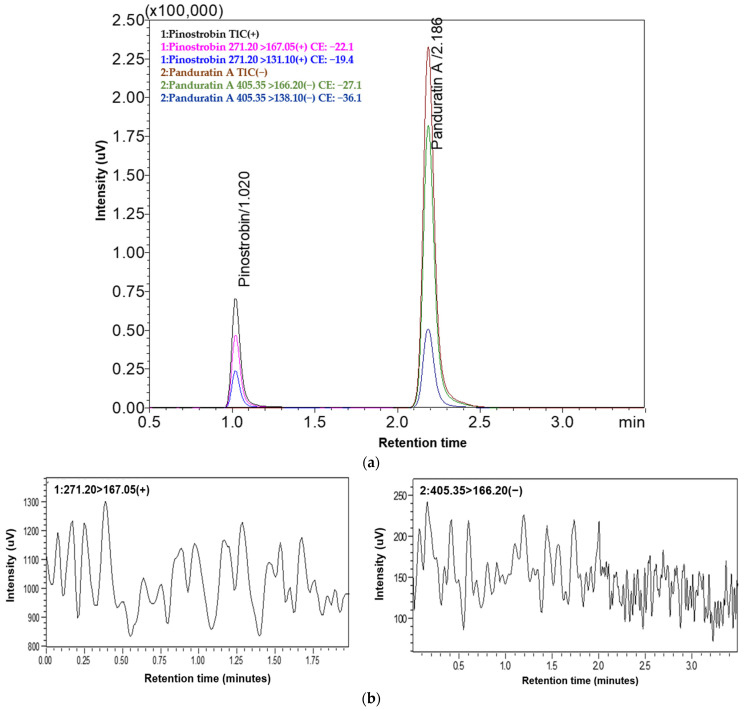
The multiple reaction monitoring (MRM) chromatogram of (**a**) standard compounds and (**b**) blank (MeOH).

This LC-ESI-MS/MS method used both quantifier (271.200→167.050; PN, 405.350→166.200; PA) and qualifier (271.200→131.100; PN, 405.35 > 138.100; PA) transitions for all quantification PN and PA of purified compounds. By establishing an intensity ratio of 30% between the two, this approach not only enhances the accuracy of PN and PA measurements, but also ensures the results are specific to the intended substances and free from contamination by other compounds. PN (C_16_H_14_O_4_) and PA (C_26_H_30_O_4_) were detected at retention times (RTs) of 1.02 and 2.18 min, respectively (Figure 2a). The molecular weight of PN was 270.28 g/mol. It ionized in the positive ESI mode and bound one H+ atom to give a parent ion *m*/*z* 271.200 [M + H]^+^. Fragmentation of the parent ion in MS2 produced fragment ions *m*/*z* 167.050 [C_8_H_7_O_4_]^+^ and 131.100 [C_9_H_7_O]^+^. PA was 406.5 g/mol, ionized in the negative ESI mode, and lost one H+ atom to give a parent ion *m*/*z* 405.350 [M-H]^−^. Fragmentation of parent ions in MS2 produced fragment ions *m*/*z* 166.200 [C_8_H_6_O_4_]^−^ and 138.100 [C_7_H_6_O_3_]^−^ (see fragmentation in Supplementary Materials). The findings agree with earlier studies that identified fragmentation patterns for analytical PN and PA that were strikingly similar [20,21,24]. In our analysis, the fragmentation of product ions in LC-ESI-MS/MS revealed two distinct cleavages of the C-ring through a retro-Diels–Alder (rDA) reaction, corroborating the observations made by Fathoni A et al. [24] and Malcolm D. McLeod et al. [25].

This validation assessed key parameters, including selectivity, linearity, precision, recovery, LOD, and LOQ. The method demonstrated a high level of selectivity, with no endogenous interference observed during the elution times of PN and PA when compared to the blank sample. This is illustrated in Figure 2, which presents the LC-ESI-MS/MS chromatograms of standard compounds (a) compared with the blank (b), highlighting the selectiveness of the method. The calibration curves for PN and PA demonstrated excellent linearity, with R^2^ values exceeding 0.996 (*n* = 3) (see calibration curves in Supplementary Materials), The LOD and LOQ were approximated by calculating from the calibration plot and were 24.025 ng/mL and 72.804 ng/mL for PN and 0.635 ng/mL and 1.924 ng/mL for PA, respectively. Both intra- and inter-batch precision and recovery studies were well within acceptable limits, with an overall %RSD of less than two for all samples. Recovery rates (based on three determinations and three replicates) ranged from 99.55 ± 1.08% to 100.38 ± 3.85% for PN and 99.65 ± 1.32% to 101.44 ± 3.05% for PA. All results are summarized in Table 2, highlighting the accuracy and precision of this analytical method.

### 2.2. Effects of Various Conditions on Extraction of PN and PA from B. rotunda by Ultrasound-Assisted Extraction and Quantification by UHPLC System

**Figure 3:**
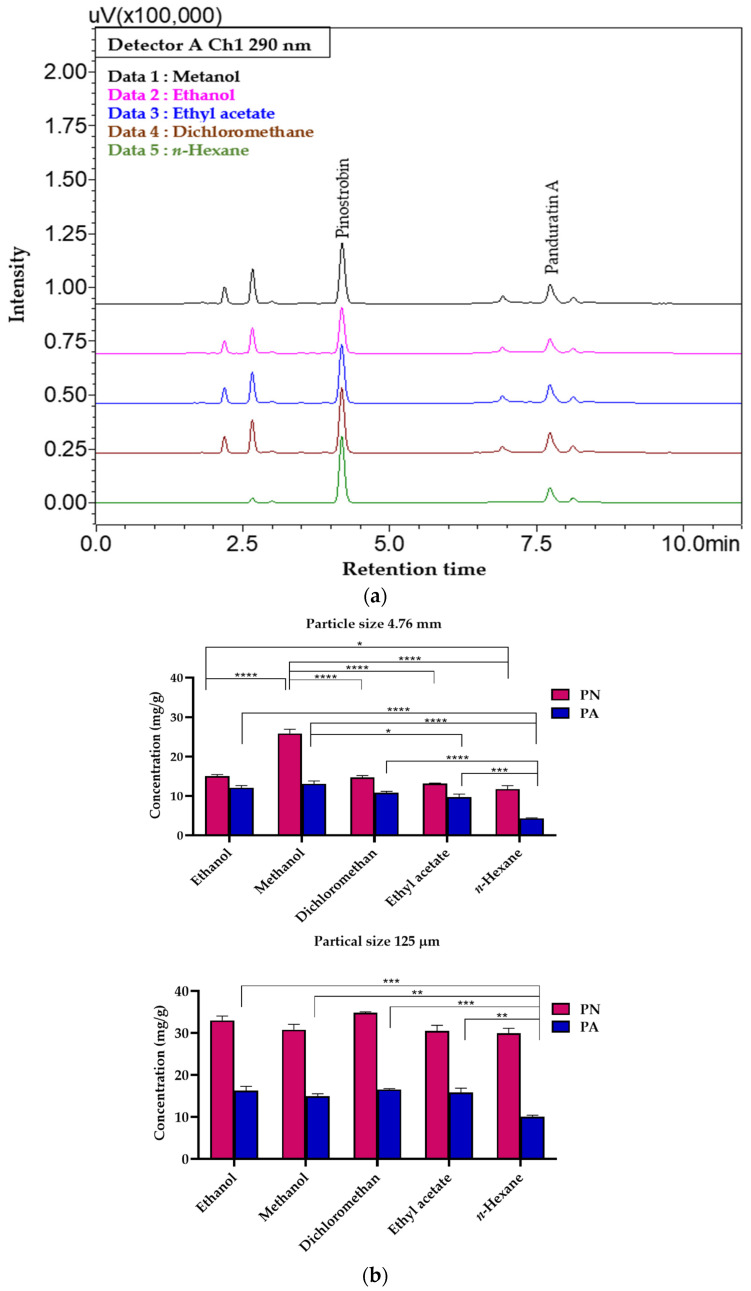
(**a**) Chromatographic profiles of extractives from *B. rotunda* using five different solvents with UAE, and (**b**) the concentrations of PN and PA in the raw material with different solvents and particle sizes. Data are the mean ± SD (*n* = 3). * *p* < 0.05, ** *p* < 0.01, *** *p* < 0.001, **** *p* < 0.0001 compared with the mean of every other parameter.

**Figure 4:**
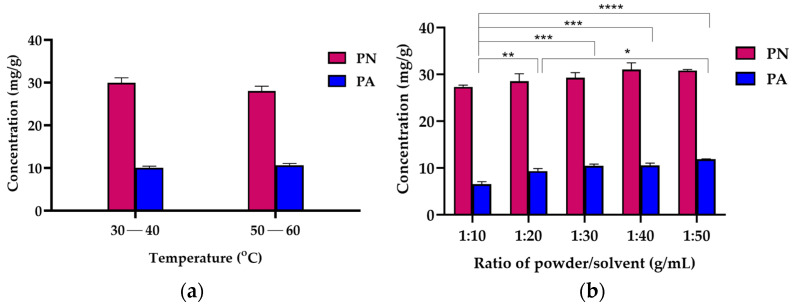

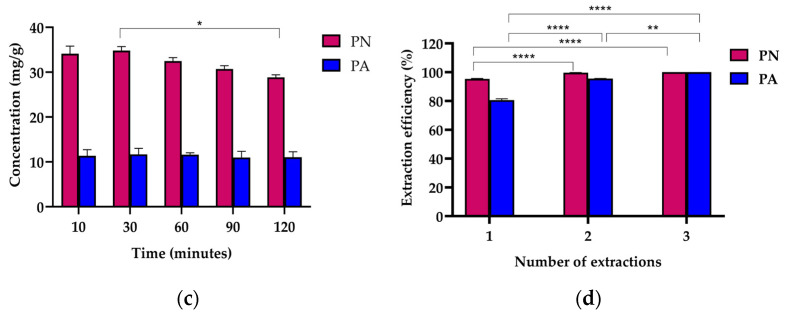
Effects of various conditions on extraction of PN and PA from *B. rotunda* (powder size 125 µm) by UAE with *n*-hexane as solvent and quantification by UHPLC system. The concentrations of PN and PA at different (**a**) extraction temperatures in range 30–40 °C and 50–60 °C, (**b**) ratios of powder/*n*-hexane in range 1:10 to 1:50 g/mL, (**c**) extraction times in range 10–120 min, and (**d**) extraction efficiencies of PN and PA at a single step compared with two and three repetitions. Data are the mean ± SD (*n* = 3). * *p* < 0.05, ** *p* < 0.01, *** *p* < 0.001, **** *p* < 0.0001 compared with the mean of every other parameter.

The ultrasound-assisted extraction (UAE) for obtaining PN and PA from the dried rhizome and root powder of *B. rotunda* was systematically conducted, focusing on various extraction parameters. Key factors such as solvent type, particle size, extraction temperature, solvent-to-material ratio, extraction duration, and repetition were evaluated for their influence on extraction yields [26], quantified by the levels of PN and PA which were extracted [27]. These findings indicate that *n*-hexane is an effective solvent for this extraction process, efficiently isolating PN and PA from *B. rotunda* while minimizing the extraction of other unwanted compounds, such as naringenin, alpinetin, pinocembrin, cardamonin, and 4-hydroxypanduratin A, which have been identified in significant amounts by Tan et al. [5] and Adhikari et al. [19]. This selective solubility highlights *n*-hexane’s utility in reducing the presence of other matrix components (Figure 3a). The particle size of the material considerably impacted the extraction yield [28], with 125 µm being identified as the optimal powder size for maximal efficiency (Figure 3b). The solvent-to-material ratio was optimized to 1:30 (g/mL), further enhancing the extraction yield (Figure 4b).

Variations in extraction temperature, time, and repetition did not significantly affect the extraction efficiency (Figure 4), suggesting that the UAE process is robust under the tested parameters.

**Figure 5:**
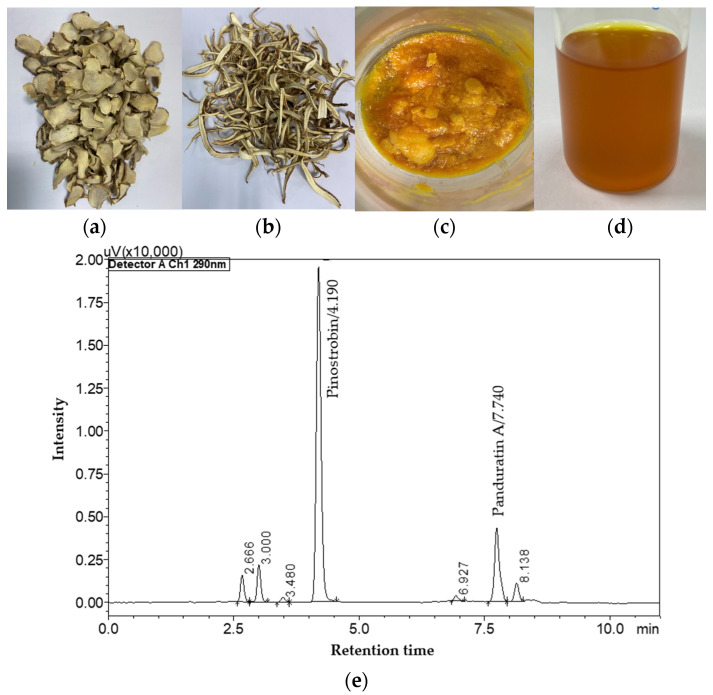
The extraction of PN and PA from *B. rotunda* using UAE with *n*-hexane solvent and quantified by UHPLC system. (**a**) Dried root, (**b**) dried rhizome, (**c**) crude *n*-hexane extract, (**d**) crude *n*-hexane extract dissolves in ethyl acetate for injection to CPC system, and (**e**) HPLC chromatogram of PN and PA from 25 µg/mL of crude *n*-hexane extract.

The UAE process utilizing *n*-hexane achieved impressive extraction yields within just 10 min, reaching a yield of 6.96 ± 0.07% (35.68 ± 1.11 mg/g for PN and 10.48 mg/g for PA, see Figure 4c). This method demonstrated an extraction efficiency of up to 95% for PN and 80% for PA with one-time extraction (Figure 4d). The low viscosity of *n*-hexane facilitates its penetration into the plant matrix, thereby enhancing the extraction process [29]. Moreover, the application of ultrasound plays a pivotal role in cell disruption and effective mass transfer, critical factors in improving extraction yields [30]. The physical characteristics of the dried raw material and the crude *n*-hexane extract, which was dissolved in ethyl acetate before injection into the CPC system, are shown in Figure 5, underlining the effectiveness of the UAE technique in isolating bioactive compounds from *B. rotunda*.

These results are consistent with previous research highlighting the advantages of UAE in extracting bioactive compounds from plant materials, showcasing its potential for maximizing yield while minimizing extraction time [31]. UHPLC chromatograms of *B. rotunda* showing the compounds obtained from the crude *n*-hexane extract are presented in Figure 5e. The chromatogram pattern corresponds to previous studies [5,19,32]. This research focuses on simple procedures that result in a high level of purity. Therefore, before purifying PN and PA from the crude extract, the sample must be sufficiently clean to facilitate easier separation using CPC. While *n*-hexane may not be the most efficient solvent for extracting large quantities of PN and PA, it is easy to evaporate and helps to produce a clean crude sample. The findings highlight the efficacy of *n*-hexane as a solvent for extracting PN and PA for separation in a single step using CPC. Nevertheless, we will continue to enhance our research by using EtOH crude extract for CPC instead of *n*-hexane, as it is a more environmentally friendly alternative.

### 2.3. Effects of Various Conditions on Separation of PN and PA from Crude n-hexane Extract by CPC System

#### 2.3.1. Effect of Partition Coefficient (K) and Separation Factor (α)

In CPC, selecting an appropriate two-phase solvent system is crucial for the effective separation of target compounds. The K values, which indicate the solubility of compounds within a biphasic solvent system, play a vital role in this process. Optimal separation typically occurs when K values range from 0.2 to 5.0. If the K value is below 0.2, no separation occurs, and the analyte remains in the mobile phase. Conversely, if the K value exceeds 5.0, the analyte is retained in the stationary phase. Thus, choosing the right solvent system is a critical step in CPC operation. Additionally, the α values, defined as the difference between the K values of the target compounds, should exceed 1.5 to 2 times the K value to ensure efficient separation [33,34,35]. In this study, the isolation of PN and PA from crude *n*-hexane extract involved using various volume ratios of two immiscible solvents, as detailed in Table 3.

**Figure 6:**
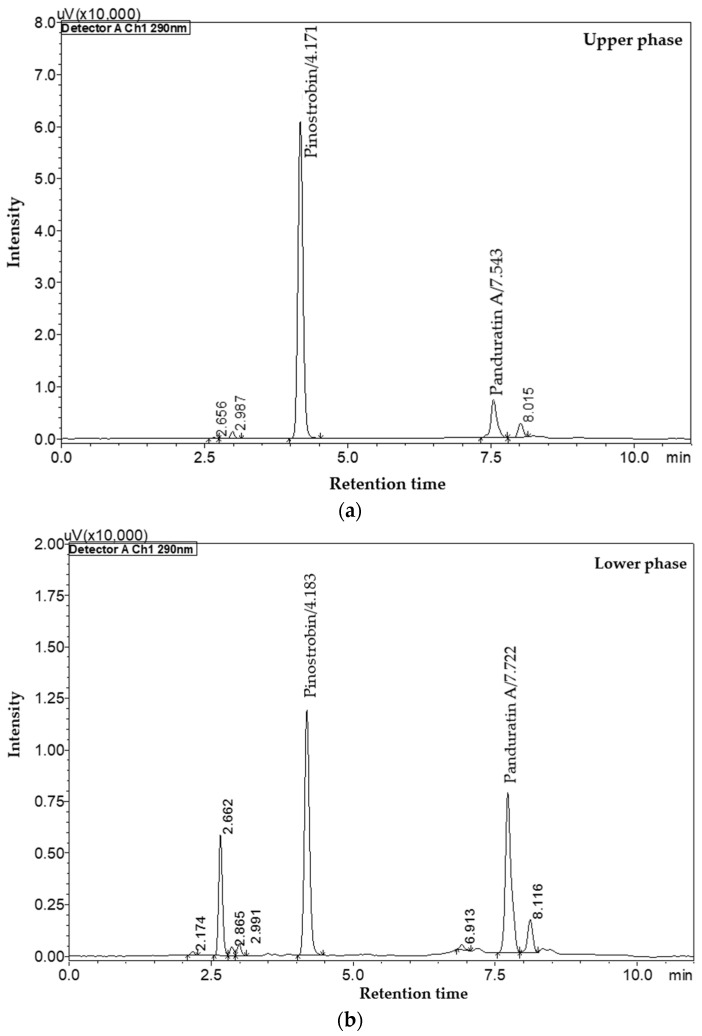
The HPLC chromatogram of PN and PA from crude *n*-hexane extract in (**a**) upper and (**b**) lower phases of *n*-hexane/MeOH/H_2_O (5/3.4/1.6, *v*/*v*) system.

The Arizona system served as a foundational springboard for the development of the solvent system in this study [36]. The solvent system consisting of *n*-hexane, methanol (MeOH), and water (H_2_O) in a ratio of 5:3.4:1.6 (*v*/*v*) produced K values of 0.2 for PN and 3.40 for PA, both within the optimal range for effective separation. The calculated α value was 17.407, indicating a high degree of selectivity between the two compounds. Although a high α value can potentially increase running times during separation, this solvent system was considered optimal because it utilizes only three solvents, compared to the four commonly used in earlier studies [37]. As illustrated in Figure 6, both PN and PA preferentially partition into the upper phase of the solvent system, reflecting their relatively non-polar characteristics. This partitioning behavior facilitates the effective segregation of other matrix components into the lower phase, allowing them to remain in the stationary phase without hindering the purification process [38]. The results highlight the efficacy of the selected solvent system in isolating PN and PA, demonstrating the advantages of CPC as a method for purifying bioactive compounds from complex matrices. These findings align with previous research showing the effectiveness of CPC in separating various phytochemicals, thereby reinforcing its application in the field of natural product extraction [38,39].

#### 2.3.2. Effects of Solvent Ratio, Sample Load Volume, and Diluent System

**Figure 7:**
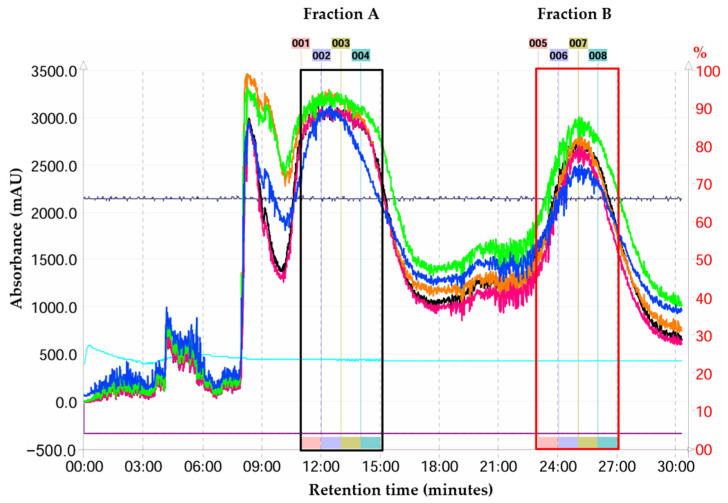
Preparative CPC (*n*-hexane: MeOH: H_2_O (5/3.4/1.6, *v*/*v*) chromatogram from crude n-hexane extract of the *B. rotunda*. The PN (Fraction A: 001–004) and PA (Fraction B: 005−008) were detected using UV detection at 220 nm (green line), 230 nm (orange line), 280 nm (pink line),285 nm (black line) and 200-600 nm (blue line).

In separation of target compounds using CPC for this experiment, the ascending mode was employed to separate PN and PA from the crude *n*-hexane extract. In this mode, the bottom aqueous phase served as the stationary phase, while the top organic phase functioned as the mobile phase. PN and PA were efficiently and straightforwardly separated from the crude *n*-hexane extract based on their optimal K values and α values. In developing a separation system using the CPC technique, the underlying principles closely resemble those of HPLC separation. Consequently, the choice of solvent system and sample conditions is crucial for achieving effective separation [36]. Therefore, this study concentrated on three primary parameters: solvent ratio, sample load volume, and diluent system, each influencing the %yield and %purity of the compounds. The optimal conditions for CPC separation of PN and PA involved using a solvent system with an *n*-hexane/MeOH/H_2_O ratio of 5:3.4:1.6 (*v*/*v*). A volume of 0.5 mL of the crude extract (1 g/mL in ethyl acetate) was added to 7 mL of the diluent system, comprising a MeOH/upper phase ratio of 3.5:3.5 mL (*v*/*v*), resulting in high-purity compounds. The extrusion process was completed in 30 min. The chromatographic analysis of the CPC fractions is presented in Figure 7, confirming the presence of PN in fraction A and PA in fraction B. Identification of these purified compounds was performed using LC-ESI-MS/MS, ensuring precise characterization of the isolated compounds. Each purified compound was weighed accurately and dissolved in MeOH at final concentrations of 500 ng/mL for PN and 250 ng/mL for PA.

**Figure 8:**
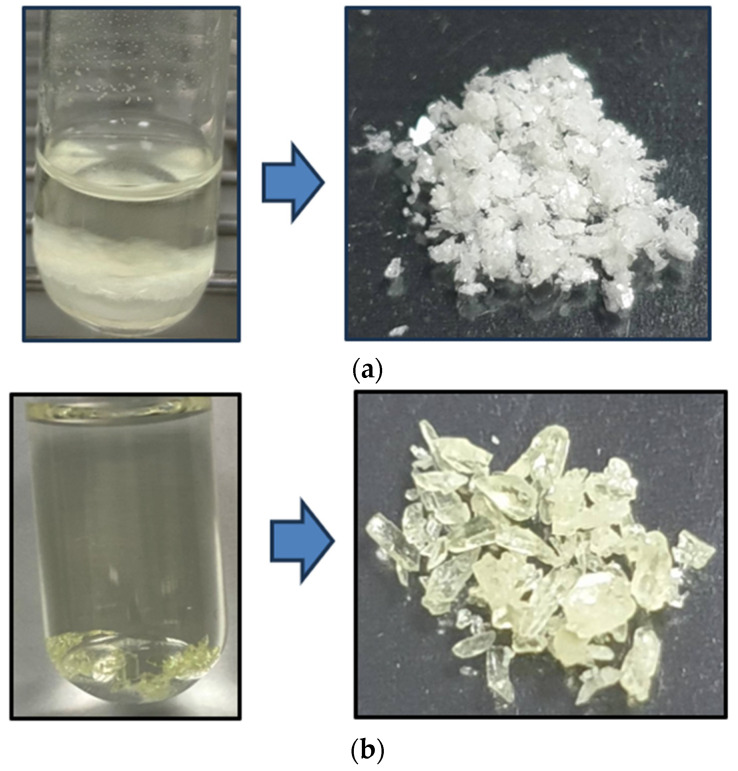
The pure crystals of isolated (**a**) PN and (**b**) PA from crude *n*-hexane extract of the *B. rotunda*.

From the crude *n*-hexane extract of B. rotunda, a total of 66.67 mg/mL, yielding 2.16 mg of PN (90% yield) and 0.4 mg of PA (57% yield), was detected by HPLC. This single-step CPC system facilitated a rapid separation process for both compounds, significantly reducing the isolation time compared to traditional methods [11,40]. Utilizing the upper phase as the mobile phase solvent allowed for the effortless evaporation of the collected fractions. The physical characteristics of the pure crystals of PN (off-white) and PA (pale yellow), depicted in Figure 8, align with previously reported findings in the literature [11,14,41], confirming the successful isolation of PN and PA from crude *n*-hexane extract of the B. rotunda. The findings from this study underscore the effectiveness of CPC as a viable method for the separation and purification of bioactive compounds from complex plant matrices, consistent with previous research highlighting the advantages of CPC in natural product isolation [42]. The yields achieved in this study align with those reported in the literature, reinforcing CPC’s potential for the efficient extraction of valuable phytochemicals [43,44].

### 2.4. Quality Assessment of Purified PN and PA

**Figure 9:**
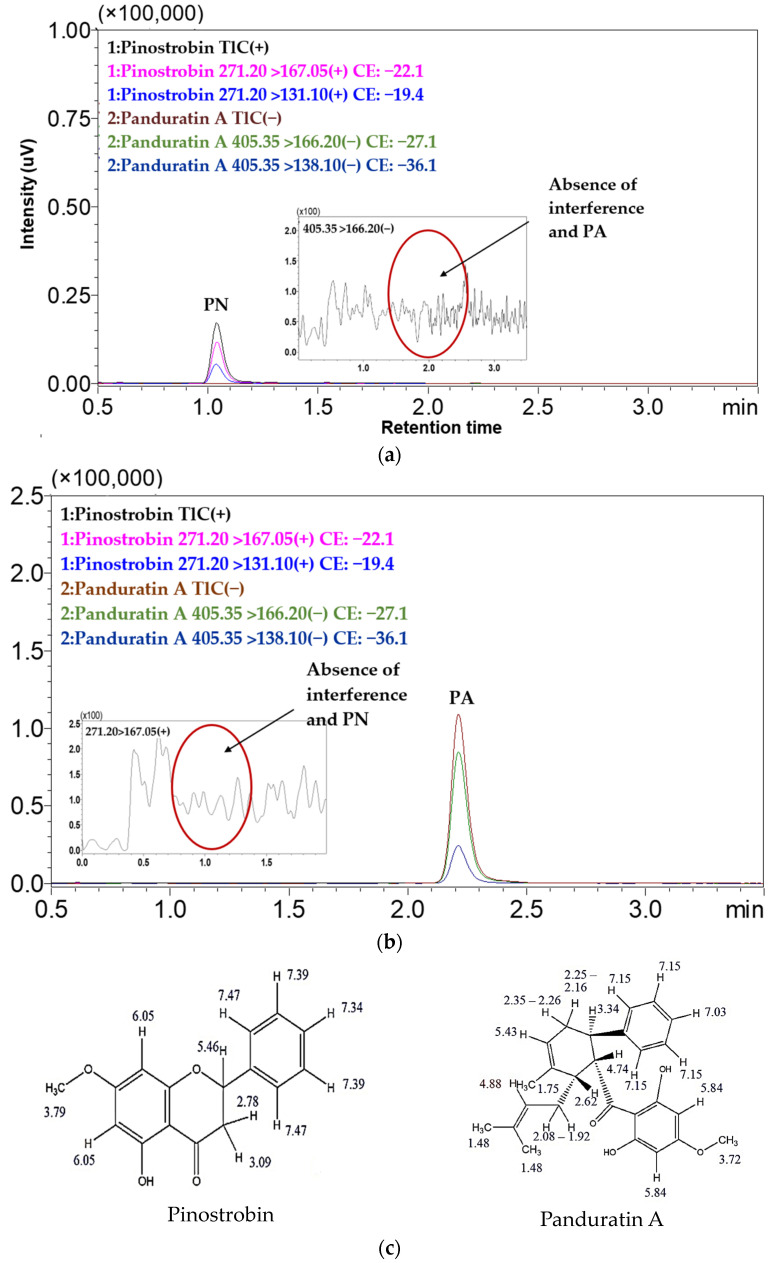
The MRM chromatogram of (**a**) PN and (**b**) PA derived from fraction A (500 ng/mL of PN) and fraction B (PN 250 of ng/mL) of separation using CPC and zoomed chromatogram. (**c**) The structures of PA and PN were further confirmed by ^1^H NMR (see Supplementary Materials).

The purified compounds PN and PA were accurately identified using LC-ESI-MS/MS. Notably, the results indicated no mutual interference between PN and PA and confirmed that each fraction of the separation was free from contamination by other substances, as illustrated in Figure 9. This absence of interference underscores the effectiveness and selectivity of the CPC method employed for the separation of these compounds [34]. The achieved purities for PA and PN were 99.69% (yielding 0.4 mg) and 98.78% (yielding 2.16 mg), respectively, as detailed in Table 4. These purity levels are significantly higher than those reported in previous studies utilizing traditional methods, which yielded purities of 96.6% for PA [11], >93.6% for PA [45], and 97.06% for PN [41]. The high purity levels obtained in this study can be attributed to the optimized conditions of the CPC method, which enable effective separation based on the partition coefficients of the target compounds. The ability of the CPC technique to achieve such high purities is consistent with findings from other studies highlighting its advantages in isolating bioactive compounds from complex mixtures. For instance, Karkoula et al. demonstrated the successful isolation of various compounds using CPC, emphasizing its efficiency in separating constituents with diverse polarities [46]. Furthermore, the use of a biphasic solvent system with appropriate partition coefficients is critical for maximizing separation efficiency. This study confirms that CPC is a highly effective method for the purification of PN and PA, providing substantial improvements in yield and purity compared to traditional extraction methods. This positions CPC as a valuable technique in the field of natural product chemistry, particularly for the isolation of bioactive compounds from herbal sources [47].

## 3. Materials and Methods

### 3.1. Plant Materials

*B. rotunda* (10–11 months) was grown at a local farm in the Mueang Nakhon Pathom district, Nakhon phathom Province, Thailand. The fresh *B. rotunda* was cleaned using running tap water and separated into two parts: roots and rhizomes. Each part of *B. rotunda* was cut with a knife into slices and dried. The dried samples were ground into powder using a stainless kitchen blender and sieved to particle sizes of 4.76 mm and 125 µm using stainless steel wire mesh to obtain a homogeneous raw material for extraction. The powder was stored in the cabinet until use in the dark at a temperature of 20 to 30 °C and 30 to 60% RH for humidity using a temperature and humidity control cabinet (WEIFO TH200, Taipei, Taiwan).

### 3.2. Reagents

Pinostrobin standard (99.95%) was purchased from the National Institute of Metrology (Pathumthani, Thailand), and Panduratin A (98.7%) standard was obtained from TLC Pharmaceutical Standards (Hyderabad, India). All solvents used for extraction and CPC separation, including *n*-hexane, dichloromethane, ethyl acetate, methanol, and ethanol, were of analytical grade and sourced from RCI Labscan. For UHPLC analysis, HPLC-grade solvents (acetonitrile and methanol) were purchased from Merck (Boston, MA, USA). Dimethyl sulfoxide, ammonium formate, and formic acid were also of analytical grade and obtained from Merck. For LC-ESI-MS/MS analysis, acetonitrile was of LC-MS grade, and ultrapure water (18.2 megohm-cm) was produced using the Barnstead™ GenPure™ Pro water purification system (Thermo Scientific, Waltham, MA, USA).

### 3.3. The Extraction of PN and PA from B. rotunda by UAE

The extraction of *B. rotunda* was performed by adding 1 g of dry powder into a test tube. The optimum conditions for the extraction of pinostrobin (PN) and panduratin A (PA) from root and rhizome powder using ultrasound-assisted extraction (UAE) were determined based on five main parameters: solvent type (ethanol, methanol (polar), dichloromethane, ethyl acetate (semi-polar), and *n*-hexane (non-polar)), extraction temperature (30–40 °C and 50–60 °C), solvent-to-sample ratio (1:10, 1:20, 1:30, 1:40, and 1:50, *v*/*v*), extraction time (10, 30, 60, 90, and 120 min), and extraction repetition (1, 2, and 3 replicates). The powder in solvent was sonicated in an ultrasonic bath (Bandelin DT1028H, Berlin, Germany) at a frequency of 35 kHz, then centrifuged, and the solvent phase was filtered through a nylon syringe filter. Finally, the solvents were removed using a vacuum evaporator (GeneVac/GENEVAC LTD./Ipswich, UK), and the residue mass was weighed for yield measurement using a 5-digit analytical balance (Sartorius, Göttingen, Germany).

### 3.4. Quantification PN and PA by UHPLC and Validation Method

The crude extract was redissolved in DMSO to approximately 10 mg/mL, and the volume was adjusted with methanol to achieve a final concentration of 25 µg/mL. This solution was filtered through a 0.22 µm nylon syringe filter, and PA and PN were quantified using a UHPLC Nexera system (Shimadzu Corporation, Kyoto, Japan). A 5 µL injection was separated using an Avantor (Radnor, PA, USA) ACE 5 µm C18 column (150 × 4.6 mm) with a Phenomenex C18 guard column (4.0 × 2.0 mm) at 40 °C. Mobile phase A consisted of 0.1% formic acid in 20 mM ammonium formate, and mobile phase B was a 50:50 (*v*/*v*) mixture of ACN and MeOH. A gradient was applied: 75% for 4.5 min, 75–90% over 0.5 min, 90–85% over 3 min, and 85–75% over 0.5 min, with a total run time of 11 min. The flow rate was 1 mL/min, and the UV detection wavelength was 290 nm [19]. The method was linear over the concentration range of 0.313–20 µg/mL for both PN and PA. Chromatographic data were evaluated using LabSolutions software version 5.65. The efficiency of extraction was determined by measuring the PA and PN content in the obtained *B. rotunda*. The UHPLC method was validated according to AOAC guidelines [17].

### 3.5. Evaluation of Partition Coefficient (K) and Separation Factor (α)

The two-phase solvent system was selected according to the K value of the target compounds in the crude extract. The K value was defined as the peak area of the target compound in the stationary phase divided by that in the mobile phase. The K value was determined using UHPLC as follows: 1 µL of 1 g/mL crude sample was added into a 2 mL tube, and then 400 µL of each phase of the pre-equilibrated two-phase solvent system was added and vigorously shaken. After the two-phase samples were thoroughly equilibrated, 200 µL of each phase was collected and subjected to UHPLC analysis. The K values for an optimum separation condition are in the following range: 0.2 ≤ K ≤ 5.0. The separation factor (α) value was the ratio of the two K values and was obtained by dividing the K values of the two compounds (α = K1/K2, where K1 > K2). The values were recommended to be >1.5.

### 3.6. Preparation of the Two-Phase Solvent System and Sample Solution for CPC System

In this study, two different biphasic solvent systems were used in the ascending mode: *n*-hexane/MeOH/H_2_O. The condition for CPC separation involved three main parameters: (1) solvent ratio: Each solvent was added to a separation funnel and shaken thoroughly. After equilibration, the upper and lower phases were separated and run into the CPC system. (2) Volume of sample load: For preparing the injection, ethyl acetate was used to dissolve the crude extract to a concentration of 1 g/mL, and 0.5, 1, 1.5, and 2 mL of crude solution were added to 7 mL of diluent. (3) Diluent system: an upper phase/lower phase ratio of 3.5/3.5 mL, *v*/*v* and a MeOH/upper phase ratio of 3.5/3.5 mL, *v*/*v* were tested for preparing the sample injection to CPC, which is an important factor in carrying the sample through the stationary phase and ensuring it has a good shape peak.

### 3.7. CPC Separation Procedure

In this experiment, an ascending mode was used for purifying compounds with CPC (model: CPC250, Gilson, Madison, WI, USA). The column was first filled with the stationary phase at a flow rate of 10 mL/min with a rotation speed of 2000 rpm (equilibrium column), and then the mobile phase was pumped into the column at the same flow rate and rotation speed (elution). After the mobile phase was flowed out of the column and a hydrostatic equilibrium was established in the column, the prepared sample solution was injected. The fractions of 10 mL were collected in test tubes by a fraction collector and monitored using a UV detector in the range of 200–600, 220, 230, 280 and 285 nm for the detection of PA and PN, with other substances included in the crude extract. Reproducible results were obtained from three repeated CPC experiments.

### 3.8. LC-ESI-MS/MS Analysis of Purified Compounds and Validation Method

Each fraction from CPC was evaporated to increase the concentration by a vacuum evaporator until the organic solvent was removed and 1 mL remained (set the program to an aqueous program and off lamp); then, the remaining solvent was frozen to be recrystallized at −20 °C. Each crystal was cleaned with cold MeOH and filtered through a nylon filter with a pore size 0.2 µm [14], weighed accurately, and dissolved in MeOH at final concentrations of 500 ng/mL and 250 ng/mL for PN and PA, respectively. They were then filtered through a nylon syringe filter with a pore size of 0.22 µm, and 1 µL of filtrated sample was injected into the LC-ESI-MS/MS system (LCMS-8040, Shimadzu Corporation, Kyoto, Japan). Chromatographic analysis was performed on Agilent (Santa Clara, CA, USA) Poroshell 120 EC-C18, 2.7 µm (3.0 × 50mm) with a Phenomenex C18 guard column (4.0 × 2.0 mm). The mobile phase A was 0.2% formic acid, and the mobile phase B was ACN at a ratio of 25:75, *v*/*v* in the isocratic elution. A flow rate of 0.5 mL/min achieved optimum separation. Nebulizing gas, heating gas, and drying gas were set at 3.0 L/min, 10.0 L/min, and 10 L/min, respectively. The DL temperature was set at 250 °C, and the heat block temperature was set at 400 °C. Multiple reaction monitoring (MRM) was performed in positive ion mode, with transitions at (*m*/*z*) 271.20→167.05 (target ion) and 271.20→131.10 (reference ion) for PN, and in negative ion mode, with transitions at 405.35→166.20 (target ion) and 405.35 > 138.10 (reference ion) for PA [48]. The target ions were used for quantification and the references were used for spectrum confirmation (reference ion ratio within ±30% [49]). The method was linear over a concentration range of 78.125 to 2500 ng/mL for PN and 15.625 to 500 ng/mL for PA. The total run time of the analysis was 4 min. Labsolutions software version 5.82 SP1 was used for the evaluation of chromatographic data. The method used for analysis was validated in accordance with AOAC guidelines [17].

### 3.9. Identified Structures of Purified Compounds by Nuclear Magnetic Resonance (^1^HNMR)

The sample was dissolved in a suitable deuterated solvent (deuterated solvents for verifying the identity of substances using NMR spectroscopy were procured from Cambridge Isotope Laboratories, Tewksbury, MA, USA). The sample was required to exhibit complete solubility without precipitate or phase separation, and it needed to be free from paramagnetic impurities. We maintained a minimum solvent level of at least 4 cm during the dissolution process. We employed an NMR tube with a standard inner diameter (i.d.) of 5 mm, ensuring it was free from chips or cracks, tightly capped, and maintained in clean conditions. Chemical structure characterization was conducted using a nuclear magnetic resonance (NMR) spectrometer on a JEOL (Peabody, MA, USA) JNM-ECZ500R/S1 spectrometer operating at 500 MHz for 1H NMR and 126 MHz for 13C NMR.

### 3.10. Statistical Analysis

The quantitative data are expressed as the mean ± standard error of the mean (SEM) from triplicate experiments. Differences between groups were determined using a one-way analysis of variance (ANOVA) followed by Tukey’s multiple comparisons test. Differences were considered significant at *p* < 0.05.

## 4. Conclusions

To the best of our knowledge, this study is the first to report the successful purification of PN and PA from the crude *n*-hexane extract of B. rotunda using centrifugal partition chromatography in ascending mode. The UHPLC-UV validation results demonstrate its potential for analyzing PN and PA in complex mixtures. The purified compounds were accurately identified via LC-ESI-MS/MS. The lack of mutual interference between PN and PA across separation fractions highlights the effectiveness and selectivity of the CPC method employed. The purities achieved for PA and PN were 99.69% and 98.78%, respectively, surpassing those obtained by traditional methods. These high purities can be attributed to the optimized CPC conditions, which utilize the partition coefficients of the target compounds for efficient separation. This study confirms that CPC is a highly effective method for purifying PN and PA, providing substantial improvements in yield and purity. The findings align with prior research that emphasizes CPC’s capabilities in isolating bioactive compounds, establishing it as a valuable technique in natural product chemistry, particularly for extracting bioactive compounds from herbal sources.

## Figures and Tables

**Table 1:** Validation parameters of UHPLC-UV method for determination of PN and PA in crude *n*-hexane extract from the *B. rotunda* at 290 nm.

Compounds	Linear Equation	R^2^	Analytical Limits		Precision (%CV)		%Recovery Range ± SD of 3 Determinations
			LOD (µg/mL)	LOQ (µg/mL)	Intra-Assay (*n* = 6, 3 Replicates)	Inter-Assay (*n* = 18, 3 Replicates)	
PN	Y = 4794.66X + 76.6758	0.9996	0.042	0.126	0.50–1.63%	1.76%	100.06 ± 0.96 to 102.19 ± 0.30
PA	Y = 3288.92X + 97.0319	0.9994	0.044	0.133	0.90–1.94%	0.60%	101.08 ± 0.49 to 102.50 ± 2.01

**Table 2:** Validation parameters of LC-ESI-MS/MS method for determination of PN and PA of purified compounds.

Compounds	Linear Equation	R^2^	Analytical Limits		Precision (%CV)		%Recovery Range ± SD of 3 Determinations
			LOD (µg/mL)	LOQ (µg/mL)	Intra-Day Assay (*n* = 6, 3 Replicates)	Inter-Day Assay (*n* = 18, 3 Replicates)	
PN	Y = 82.5225X + 1066.06	0.9998	24.025	72.804	1.71–1.93%	1.56%	99.555 ± 1.08 to 100.38 ± 3.85
PA	Y = 2060.46X + 5618.97	0.9969	0.635	1.924	1.11–1.58%	0.83%	99.649 ± 1.317 to 101.44 ± 3.05

**Table 3:** K values and α values of compounds in different solvent systems.

Solvent System	Ratio (*v*/*v*)	Solubility of PN in Solvent System (Area)		K_1_	Solubility of PA in Solvent System (Area)		K_2_	α
		Upper	Lower		Upper	Lower		
*n*-hexane/MeOH/H_2_O	5/3.2/1.8	324,352	52,725	0.16	14,469	42,017	2.90	17.86
	5/3.3/1.7	323,672	46,876	0.15	14,942	38,041	2.55	17.58
	5/3.4/1.6	374,084	73,138	0.20	16,140	54,928	3.40	17.41
	5/3.5/1.5	304,702	79,302	0.26	12,067	57,781	4.79	18.40

**Table 4:** The amount of pure crystal, %yield, and %purity of PN and PA isolated by CPC.

Compounds	Calculated Compounds in Crude *n*-hexane Extract (mg)	Obtained Pure Crystals (mg)	%Yield	%Purity
PN	~2.4 mg	2.16 ± 0.11	90%	98.78 ± 4.78
PA	~0.7 mg	0.4 ± 0.02	57%	99.69 ± 4.98

## Data Availability

Data are contained within the article and Supplementary Materials.

## Supplementary Materials

The following supporting information can be downloaded at: https://www.mdpi.com/article/10.3390/molecules29215186/s1. Figure S1: Linearity of PN and PA standard at range 0.313-20 µg/ml (n=3) of UHPLC-UV method. Figure S2:UV-UHPLC chromatogram of PN and PA from crude extract when samples were analyzed consecutively. Figure S3: Linearity of compounds standard at range 78.125-2,500 ng/ml for PN and 15.625-500 for PA(n=3). Figure S4: The chemical structures and mass spectra of (a) PN and (b) PA. Figure S5: Overlaid LC-ESI/MS/MS chromatogram of PN and PA standard compared with derived from fractions A(500 ng/ml of PN) and fractions B (PN 250 of ng/ml) of separation using CPC. Figure S6: UV-UHPLC chromatogram of PN and PA derived from separation using CPC. Figure S7: ^1^H NMR spectrum of (a) PN and (b) PA.

## References

[1] Wang P., Wen C., Olatunji O.J. (2022). Anti-Inflammatory and Antinociceptive Effects of *Boesenbergia rotunda* Polyphenol Extract in Diabetic Peripheral Neuropathic Rats. J. Pain. Res..

[2] Chitapanarux T., Lertprasertsuke N., Toworakul C. (2021). Efficacy and safety of fingerroot (*Boesenbergia rotunda*) extract in patients with functional dyspepsia: A randomized, placebo-controlled trial. Digestion.

[3] Eakwaropas P., Aye N.M.M., Ngawhirunpat T., Pamornpathomkul B. (2022). Antioxidant Activity, Antimicrobial Activities, and Effect on the Viability of Fibroblast Cells of *Boesenbergia rotunda*-Loaded Hydrogel Patches. Key Eng. Mater..

[4] Md-Mustafa N.D., Khalid N., Gao H., Peng Z., Alimin M.F., Bujang N., Ming W.S., Mohd-Yusuf Y., Harikrishna J.A., Othman R.Y. (2014). Transcriptome profiling shows gene regulation patterns in a flavonoid pathway in response to exogenous phenylalanine in *Boesenbergia rotunda* cell culture. BMC Genom..

[5] Tan B.C., Tan S.K., Wong S.M., Ata N., Rahman N.A., Khalid N. (2015). Distribution of Flavonoids and Cyclohexenyl Chalcone Derivatives in Conventional Propagated and In Vitro-Derived Field-Grown *Boesenbergia rotunda* (L.). Mansf. Evid. -Based Complement. Altern. Med..

[6] Sujana D., Sumiwi S.A., Saptarini N.M., Levita J. (2024). The Nephroprotective Activity of *Boesenbergia rotunda* Rhizome by Reducing Creatinine, Urea Nitrogen, Glutamic Pyruvic Transaminase, and Malondialdehyde Levels in the Blood and Attenuating the Expression of Havcr1 (KIM-1), Lcn2 (NGAL), Casp3, and Casp7 Genes in the Kidney Cortex of Cisplatin-Induced Sprague-Dawley Rats. J. Exp. Pharmacol..

[7] Yu M., Gouvinhas I., Rocha J., Barros A.I. (2021). Phytochemical and antioxidant analysis of medicinal and food plants towards bioactive food and pharmaceutical resources. Sci. Rep..

[8] Wang T., Liu C., Shu S., Zhang Q., Olatunji O.J. (2022). Therapeutic efficacy of polyphenol-rich fraction of *Boesenbergia rotunda* in diabetic rats: A focus on hypoglycemic, antihyperlipidemic, carbohydrate metabolism, antioxidant, anti-inflammatory and pancreato-protective activities. Front. Biosci.-Landmark.

[9] Kim D.-Y., Kim M.-S., Sa B.-K., Kim M.-B., Hwang J.-K. (2012). Boesenbergia pandurata attenuates diet-induced obesity by activating AMP-activated protein kinase and regulating lipid metabolism. Int. J. Mol. Sci..

[10] Zhang L., Jiang Q., Wang X., Jaisi A., Olatunji O.J. (2023). *Boesenbergia rotunda* displayed anti-inflammatory, antioxidant and anti-apoptotic efficacy in doxorubicin-induced cardiotoxicity in rats. Sci. Rep..

[11] Salama S.M., AlRashdi A.S., Abdulla M.A., Hassandarvish P., Bilgen M. (2013). Protective activity of Panduratin A against thioacetamide-induced oxidative damage: Demonstration with in vitro experiments using WRL-68 liver cell line. BMC Complement. Altern. Med..

[12] Abdelwahab S.I., Mohan S., Abdulla M.A., Sukari M.A., Abdul A.B., Taha M.M.E., Syam S., Ahmad S., Lee K.-H. (2011). The methanolic extract of *Boesenbergia rotunda* (L.) Mansf. and its major compound pinostrobin induces anti-ulcerogenic property in vivo: Possible involvement of indirect antioxidant action. J. Ethnopharmacol..

[13] Eng-Chong T., Yean-Kee L., Chin-Fei C., Choon-Han H., Sher-Ming W., Li-Ping C.T., Gen-Teck F., Khalid N., Abd Rahman N., Karsani S.A. (2012). *Boesenbergia rotunda*: From ethnomedicine to drug discovery. Evid. -Based Complement. Altern. Med..

[14] Fakhrudin N., Pramiastuti O., Wahyuono S. (2021). A simple, fast, and inexpensive nonchromatographic method for the isolation of pinostrobin from *Boesenbergia rotunda* rhizome. Rasayan J. Chem..

[15] Kumar B.R. (2017). Application of HPLC and ESI-MS techniques in the analysis of phenolic acids and flavonoids from green leafy vegetables (GLVs). J. Pharm. Anal..

[16] Mishra J., Sharma R.K., Misra K. (2018). Characterization techniques for herbal products. Management of High Altitude Pathophysiology.

[17] International A. (2013). Appendix K: Guidelines for dietary supplements and botanicals. AOAC Off. Methods Anal..

[18] Vassiliadis S., Elkins A.C., Reddy P., Guthridge K.M., Spangenberg G.C., Rochfort S.J. (2019). A simple LC–MS method for the quantitation of alkaloids in endophyte-infected perennial ryegrass. Toxins.

[19] Adhikari D., Gong D.-S., Oh S.H., Sung E.H., Lee S.O., Kim D.-W., Oak M.-H., Kim H.J. (2020). Vasorelaxant effect of *Boesenbergia rotunda* and its active ingredients on an isolated coronary artery. Plants.

[20] Kim M., Choi S., Noh K., Kim C., Kim E., Hwang J.K., Kang W. (2017). Determination of panduratin A in rat plasma by HPLC-MS/MS and its application to a pharmacokinetic study. J. Pharm. Biomed. Anal..

[21] Sun X., Liu X., Chen S. (2020). The pharmacokinetics, tissue distribution, metabolism, and excretion of pinostrobin in rats: Ultra-high-performance liquid chromatography coupled with linear trap quadrupole orbitrap mass spectrometry studies. Front. Pharmacol..

[22] Shrivastava A., Gupta V.B. (2011). Methods for the determination of limit of detection and limit of quantitation of the analytical methods. Chron. Young Sci..

[23] Titier K., Picard S., Ducint D., Teilhet E., Moore N., Berthaud P., Mahon F.-X., Molimard M. (2005). Quantification of imatinib in human plasma by high-performance liquid chromatography-tandem mass spectrometry. Ther. Drug Monit..

[24] Fathoni A., Candraditya A., Rudiana T. (2022). Antioxidant activity and identification of flavonoid compounds in Patat. J. Pendidik. Kim..

[25] Pasfield L.A., de la Cruz L., Ho J., Coote M.L., Otting G., McLeod M.D. (2013). Synthesis of (±)-panduratin A and related natural products using the high pressure Diels–Alder reaction. Asian J. Org. Chem..

[26] Ponphaiboon J., Krongrawa W., Aung W.W., Chinatangkul N., Limmatvapirat S., Limmatvapirat C. (2023). Advances in Natural Product Extraction Techniques, Electrospun Fiber Fabrication, and the Integration of Experimental Design: A Comprehensive Review. Molecules.

[27] Liana D., Eurtivong C., Phanumartwiwath A. (2024). *Boesenbergia rotunda* and Its Pinostrobin for Atopic Dermatitis: Dual 5-Lipoxygenase and Cyclooxygenase-2 Inhibitor and Its Mechanistic Study through Steady-State Kinetics and Molecular Modeling. Antioxidants.

[28] Zhang Y., Liu C., Li J., Qi Y., Li Y., Li S. (2015). Development of “ultrasound-assisted dynamic extraction” and its combination with CCC and CPC for simultaneous extraction and isolation of phytochemicals. Ultrason. Sonochem..

[29] Wang S., Lin A.H.-M., Han Q., Xu Q. (2020). Evaluation of direct ultrasound-assisted extraction of phenolic compounds from potato peels. Processes.

[30] Phaiphan A., Baharin B.S. (2019). Ultrasound-assisted extraction produce better antibacterial and antioxidant activities of *Senna siamea* (Lam.) leaf extracts than solvent extraction. Malays. J. Microbiol..

[31] Hao C., Chen L., Dong H., Xing W., Xue F., Cheng Y. (2020). Extraction of flavonoids from scutellariae radix using ultrasound-assisted deep eutectic solvents and evaluation of their anti-inflammatory activities. ACS Omega.

[32] Yusuf N.A., M Annuar M.S., Khalid N. (2013). Existence of bioactive flavonoids in rhizomes and plant cell cultures of ‘*Boesenbergia rotund*’ (L.). Mansf. Kulturpfl. Aust. J. Crop Sci..

[33] Kim H.-H., Kim H.-S., Ko J.-Y., Kim C.-Y., Lee J.-H., Jeon Y.-J. (2016). A single-step isolation of useful antioxidant compounds from Ishige okamurae by using centrifugal partition chromatography. Fish. Aquat. Sci..

[34] Nam B., Paudel S.B., Kim J.-B., Jin C.H., Lee D., Nam J.-W., Han A.-R. (2019). Preparative Separation of Three Monoterpenes from Perilla frutescens var. crispa using centrifugal partition chromatography. Int. J. Anal. Chem..

[35] Liakakou A., Angelis A., Papachristos D.P., Fokialakis N., Michaelakis A., Skaltsounis L.A. (2021). Isolation of volatile compounds with repellent properties against Aedes albopictus (Diptera: Culicidae) using CPC technology. Molecules.

[36] Boonloed A., Weber G.L., Ramzy K.M., Dias V.R., Remcho V.T. (2016). Centrifugal partition chromatography: A preparative tool for isolation and purification of xylindein from *Chlorociboria aeruginosa*. J. Chromatogr. A.

[37] Lorántfy L., Rutterschmid D., Örkényi R., Bakonyi D., Faragó J., Dargó G., Könczöl Á. (2020). Continuous Industrial-Scale Centrifugal Partition Chromatography with Automatic Solvent System Handling: Concept and Instrumentation. Org. Process Res. Dev..

[38] Spînu S., Ortan A., Ionescu D., Moraru I. (2020). Centrifugal partition chromatography (CPC)–A novel method of separation and purification of natural products-a SHORT review. Curr. Trends Nat. Sci..

[39] Kim H.-S., Fernando I.P.S., Lee S.-H., Ko S.-C., Kang M.C., Ahn G., Je J.-G., Sanjeewa K., Rho J.-R., Shin H.J. (2021). Isolation and characterization of anti-inflammatory compounds from Sargassum horneri via high-performance centrifugal partition chromatography and high-performance liquid chromatography. Algal Res..

[40] Nyokat N., Yen K.H., Hamzah A.S., Lim I.F., Saaidin A.S. (2017). Isolation and synthesis of pinocembrin and pinostrobin from *Artocarpus odoratissimus*. Malays. J. Anal. Sci..

[41] Pattamadilok D., Sakpetch A. (2021). Isolation of pinostrobin, a chemical marker from fingerroots for quality control purposes. J. Tradit. Thai Altern. Med..

[42] Kim H.-S., Je J.-G., An H., Baek K., Lee J.M., Yim M.-J., Ko S.-C., Kim J.-Y., Oh G.-W., Kang M.-C. (2022). Isolation and characterization of efficient active compounds using high-performance centrifugal partition chromatography (CPC) from anti-inflammatory activity fraction of *Ecklonia maxima* in South Africa. Mar. Drugs.

[43] Correa D.I., Pastene-Navarrete E., Bustamante L., Baeza M., Alarcón-Enos J. (2020). Isolation of Three Lycorine Type Alkaloids from *Rhodolirium speciosum* (Herb.) Ravenna Using pH-Zone-Refinement Centrifugal Partition Chromatography and Their Acetylcholinesterase Inhibitory Activities. Metabolites.

[44] Maly M., Benes F., Binova Z., Zlechovcova M., Kastanek P., Hajslova J. (2023). Effective isolation of cannabidiol and cannabidiolic acid free of psychotropic phytocannabinoids from hemp extract by fast centrifugal partition chromatography. Anal. Bioanal. Chem..

[45] Kongratanapasert T., Boonyarattanasoonthorn T., Supannapan K., Hongeng S., Khemawoot P. (2024). Oral Bioavailability, Tissue Distribution, Metabolism, and Excretion of Panduratin A from *Boesenbergia rotunda* Extract in Healthy Rats. Drug Des. Dev. Ther..

[46] Karkoula E., Angelis A., Koulakiotis N.S., Gikas E., Halabalaki M., Tsarbopoulos A., Skaltsounis A.L. (2018). Rapid isolation and characterization of crocins, picrocrocin, and crocetin from saffron using centrifugal partition chromatography and LC–MS. J. Sep. Sci..

[47] Bojczuk M., Żyżelewicz D., Hodurek P. (2017). Centrifugal partition chromatography–A review of recent applications and some classic references. J. Sep. Sci..

[48] Boonyarattanasoonthorn T., Kongratanapasert T., Jiso A., Techapichetvanich P., Nuengchamnong N., Supannapan K., Kijtawornrat A., Khemawoot P. (2023). Absolute oral bioavailability and possible metabolic pathway of panduratin A from *Boesenbergia rotunda* extract in beagle dogs. Pharm. Biol..

[49] Goshawk J., Wood M. Evaluation of Ion Ratios as an Additional Level of Confirmation in Accurate Mass Toxicology Screening. Waters White Paper 720005866. https://www.waters.com/waters/library.htm?locale=en_US&lid=134922443&srsltid=AfmBOop7R_Gf6nbsYWAc0iJoGdB9Ifc5TxpI3qlvZdb20Ma1ESgZgugW.

