# Supplementary material for: Efficient Simultaneous Isolation of Pinostrobin and Panduratin A from Boesenbergia rotunda Using Centrifugal Partition Chromatography
Source: Molecules. 2024 Nov 2;29(21):5186. doi: 10.3390/molecules29215186 (PMC11547732; doi:10.3390/molecules29215186)
Supplement: Supplementary file 1 [file molecules-29-05186-s001.zip › molecules-3271204-supplementary.pdf]

Supplementary material: ID molecules-3271204

1. Linearity for UV-UHPLC method.

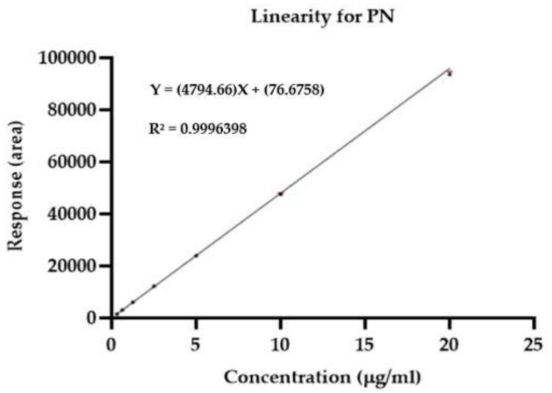

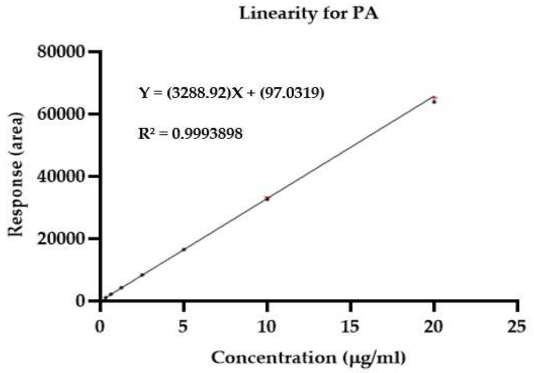

Figure S1. Linearity of PN and PA standard at range 0.313-20 μg/ml (n=3) of UV-UHPLC method.

2. UV-UHPLC chromatogram of PN and PA from crude extract when were analyzed consecutively (n=6).

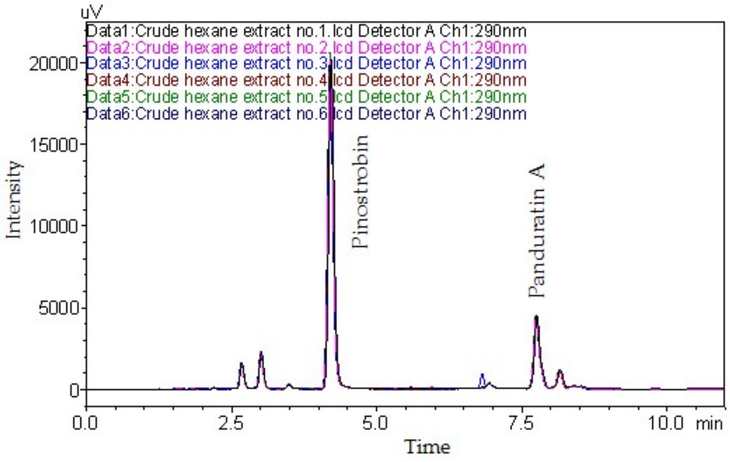

Figure S2 UV-UHPLC chromatogram of PN and PA from crude extract when samples were analyzed consecutively.

3. Linearity for LC-ESI/MS/MS method.

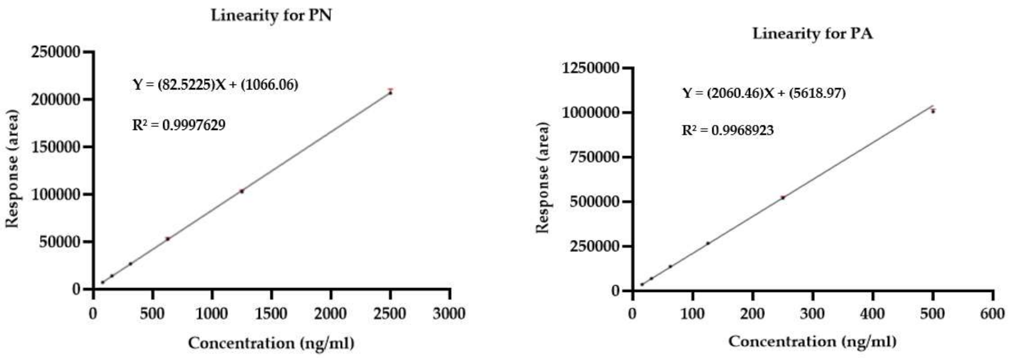

Figure S3. Linearity of compounds standard at range 78.125-2,500 ng/ml for PN and 15.625-500 for PA(n=3).

3. Fragmentation of compounds in LC-ESI/MS/MS.

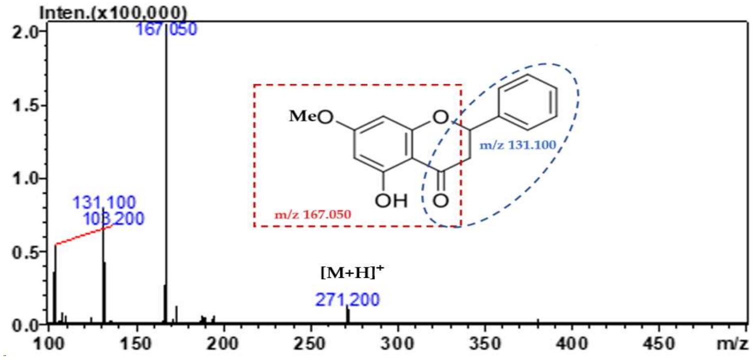

(a)

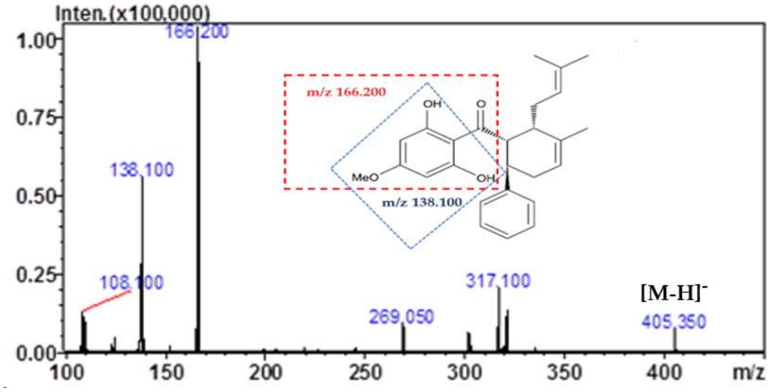

(b)

Figure S4. The chemical structures and mass spectra of (a) PN and (b)

PA.

4. Identification of purified compounds.

4.1 Identification of purified compounds using LC-ESI/MS/MS method

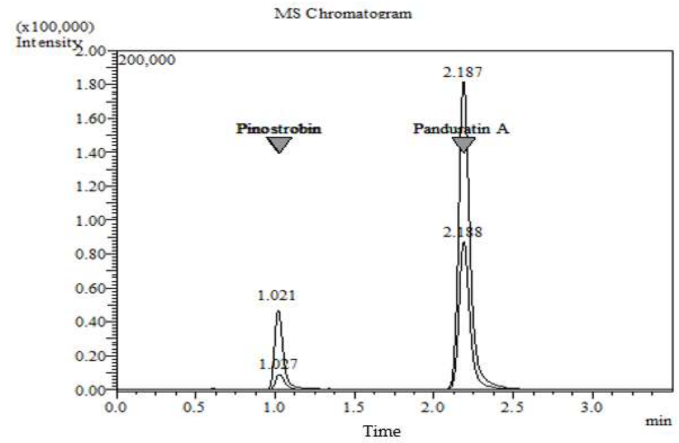

Figure S5. Overlaid LC-ESI/MS/MS chromatogram of PN and PA standard compared with derived from fractions A(500 ng/ml of PN) and fractions B (PN 250 of ng/ml) of separation using CPC.

4.2 Identification of purified compounds using UV-UHPLC method

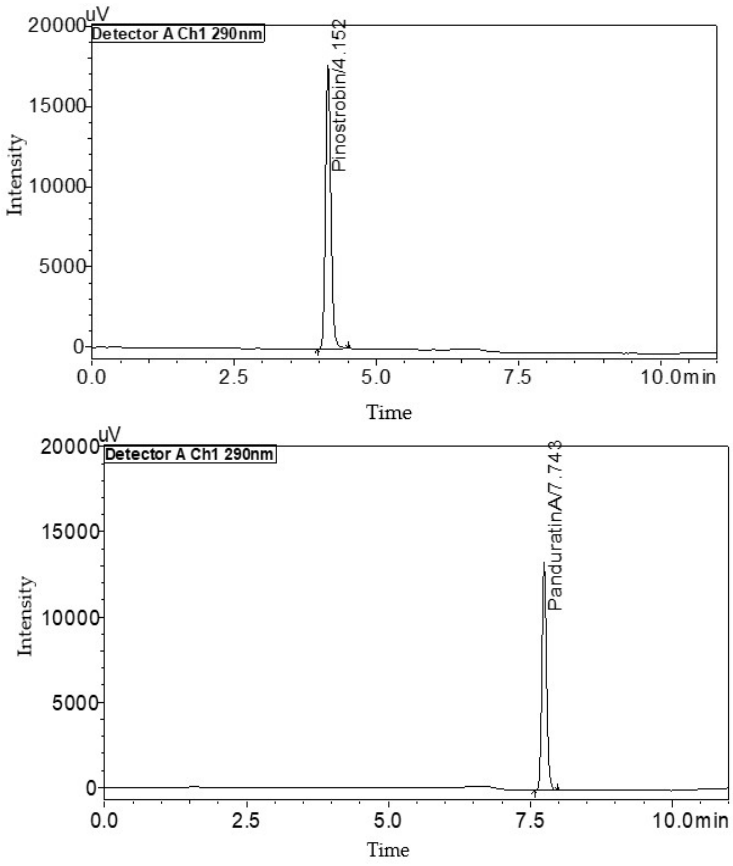

Figure S6 UV-UHPLC chromatogram of PN and PA derived from separation using CPC.

4.3 Structural identification of purified compounds by nuclear magnetic resonance (^{1}HNMR)

The chemical structures of the target compounds were determined using ^{1}H NMR spectroscopy, with the NMR data presented as follows. The ^1H NMR spectrum data for PN was recorded at 500 MHz in METHANOL-D4 and showed δ 7.51–7.42 (m, 2H), 7.44–7.26 (m, 3H), 6.05 (dt, J = 17.7, 2.2 Hz, 2H), 5.46 (dd, J = 12.8, 3.1 Hz, 1H), 3.79 (d, J = 1.7 Hz, 3H), 3.09 (dd, J = 17.1, 12.8 Hz, 1H), and 2.78 (dd, J = 17.1, 3.1 Hz, 1H).

For PA, the ^{1}H NMR spectrum data (500 MHz, METHANOL-D4) were δ 7.15 (d, J = 4.4 Hz, 4H), 7.03 (h, J = 4.1 Hz, 1H), 5.84 (s, 2H), 5.39 (d, J = 2.2 Hz, 1H), 4.73 (dd, J = 11.6, 4.7 Hz, 1H), 3.73 (s, 3H), 3.34 (d, J = 6.5 Hz, 1H), 2.62 (dt, J = 8.2, 4.7 Hz, 1H), 2.36–2.28 (m, 1H), 2.27–2.17 (m, 1H), 2.08–1.90 (m, 2H), 1.75 (q, J = 1.8 Hz, 3H), and 1.48 (dd, J = 6.2, 1.3 Hz, 6H). The results are illustrated in Figure 7.

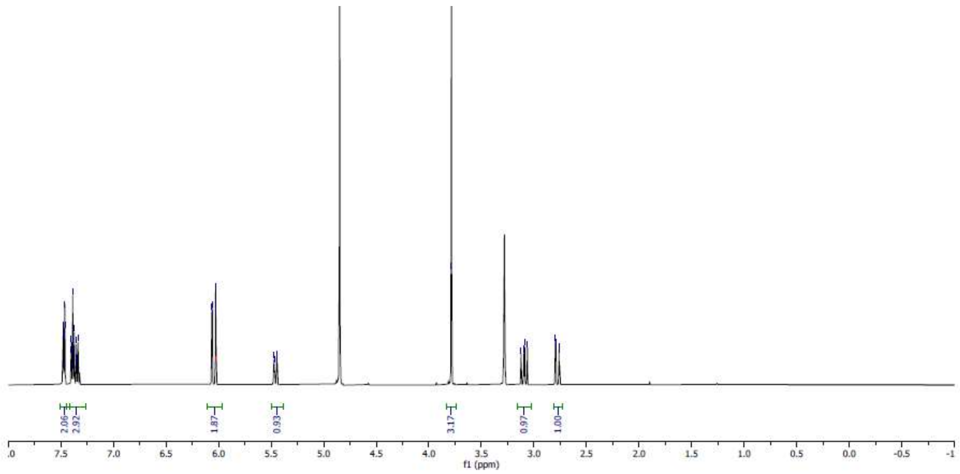

(a)

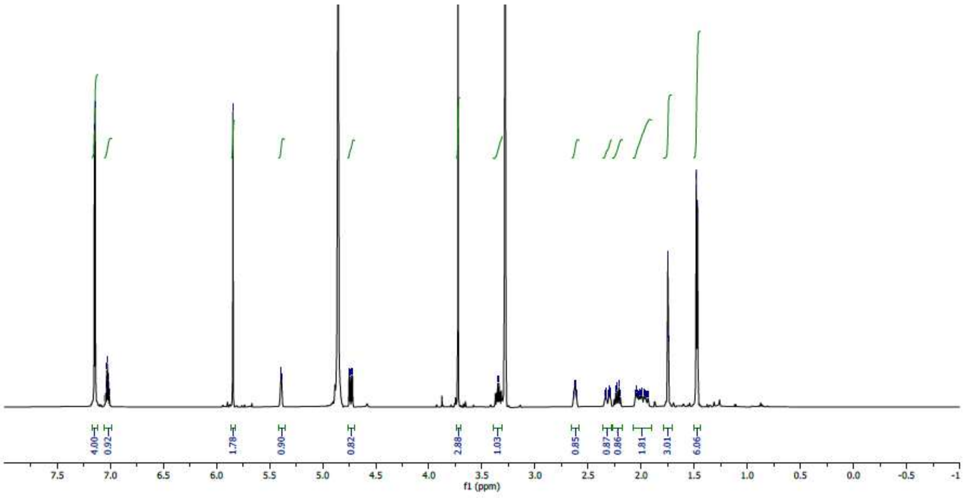

(b)

Figure S7. ^{1}H NMR spectrum of (a) PN and (b) PA.
